# Supplementary material for: Two noncompeting human neutralizing antibodies targeting MPXV B6 show protective effects against orthopoxvirus infections
Source: Nat Commun. 2024 May 31;15:4660. doi: 10.1038/s41467-024-48312-2 (PMC11143242; doi:10.1038/s41467-024-48312-2)
Supplement: Supplementary file 1 — Supplementary information [file 41467_2024_48312_MOESM1_ESM.pdf]

Supplementary information for

Two noncompeting human neutralizing antibodies targeting MPXV B6 show protective effects against orthopoxvirus infections

Runchu Zhao[1#], Lili Wu[1#], Junqing Sun[1,2#], Dezhi Liu[1,3#], Pu Han[1], Yue Gao[1,4], Yi Zhang[1,3], Yanli Xu[5], Xiao Qu[1], Han Wang[6], Yan Chai[1], Zhihai Chen[5], George F. Gao[1,2,7], Qihui Wang[1,3,7*]

[#] These authors contributed equally to this work.

[*] Correspondence: wangqihui@im.ac.cn (Q.W.)

This file includes:

Supplementary Figures 1-6

Supplementary Table 1

Supplementary Figure 1

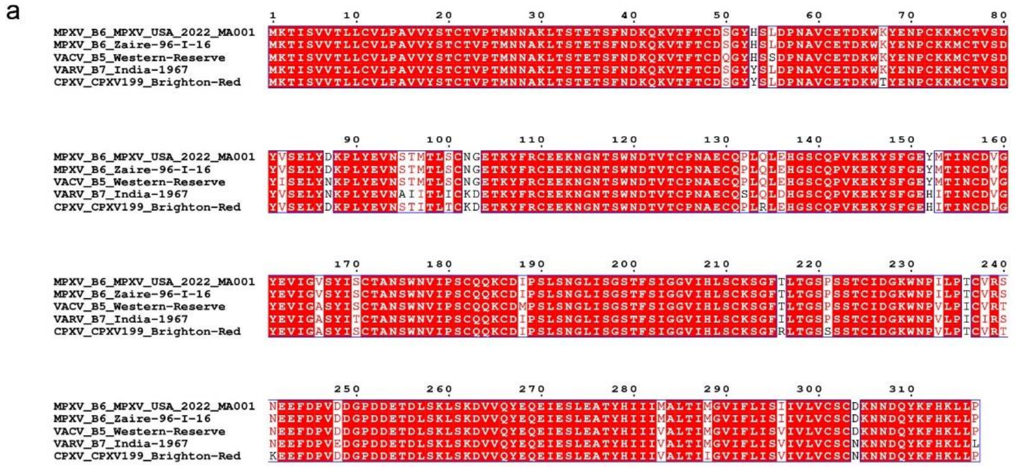

b

Homology	MPXV_B6 (MPXV_USA_2022_MA001)	MPXV_B6 (Zaire-96-I-16)	VACV_B5 (Western Reserve)	VARV_B7 (India-1967)	CPXV_CPXV199 (Brighton Red)
MPXV_B6 (MPXV_USA_2022_MA001)	100%				
MPXV_B6 (Zaire-96-I-16)	100%	100%			
VACV_B5 (Western Reserve)	96.53%	96.53%	100%		
VARV_B7 (India-1967)	93.04%	93.04%	93.35%	100%	
CPXV_CPXV199 (Brighton Red)	93.69%	93.69%	93.38%	94.30%	100%

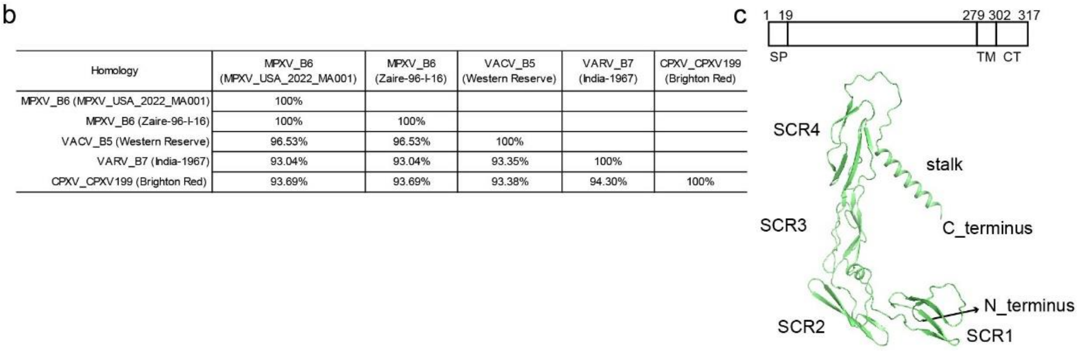

Supplementary Figure 1 Conservative analysis of B6 among orthopoxvirus. a and **b** Sequence alignment (**a**) and identity (**b**) of MPXV B6 from clade II (MPXV_USA_2022_MA001 strain, accession no. URK20605.1) and clade I (Zaire-96-I-16 strain, accession no. NP_536594.1) and its orthologs in VACV (Western Reserve strain, accession no. YP_233069.1), VARV (India-1967 strain, accession no. NP_042219.1) and CPXV (Brighton Red strain, accession no. NP_619980.1). **c** Schematic representation of B6 protein and its structure predicted by using AlphaFold2.

Supplementary Figure 2

	V allele	D allele	J allele	CDR1	CDR2	CDR3
hMB621_H	IGHV3-30*04 (93.9%)	IGHD2-2*02 (100%)	IGHJ4*03 (97.1%)	GFSISTYP	ISHDGRNK	ARAYPYAFDV
hMB621_K	IGKV3-15*01 (94.8%)	-	IGKJ1*01 (100%)	QSVRND	GAS	QQYKDWPPWT
hMB668_H	IGHV1-69*09 (93.5%)	IGHD3-3*02 (100%)	IGHJ6*01 (93.8%)	GGTFSDYA	ILPIVGVP	ARRSGINGHGLDV
hMB668_K	IGKV2-28*01 (95.0%)	-	IGKJ2*01 (97.4%)	QSLLNTNGYNY	LGS	MQTLQTQGYT

Supplementary Figure 2 Germline analysis of hMB621 and hMB668. The top matches to *Homo sapiens* germline genes and the nucleotide sequence identities of V-(D)-J of hMB621 and hMB668 were listed. CDR1, CDR2 and CDR3 indicated the amino acid sequences of CDR for both H and L chains. The amino acids in red color indicated the somatic hypermutation comparing to their germline sequences.

Supplementary Figure 3

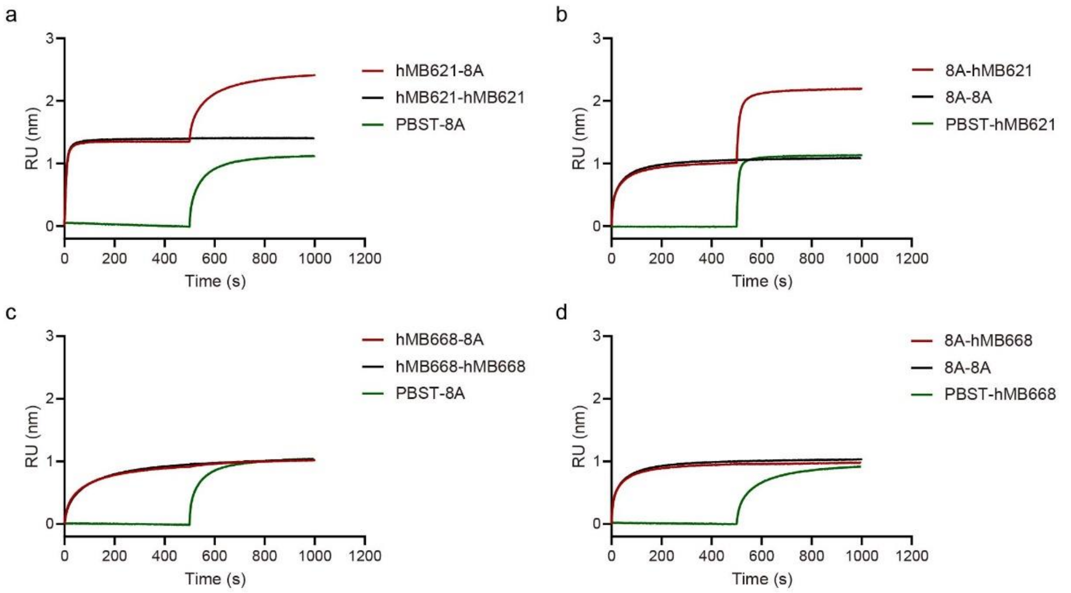

Supplementary Figure 3 Competitive binding of hMB621 and hMB668 with 8A on MPXV B6 measured by Octet RED96. a and **b** Competitive binding of hMB621 with 8A on MPXV B6. **c** and **d** Competitive binding of hMB668 with 8A on MPXV B6. The assay was performed twice. Shown data were one representative result.

Supplementary Figure 4

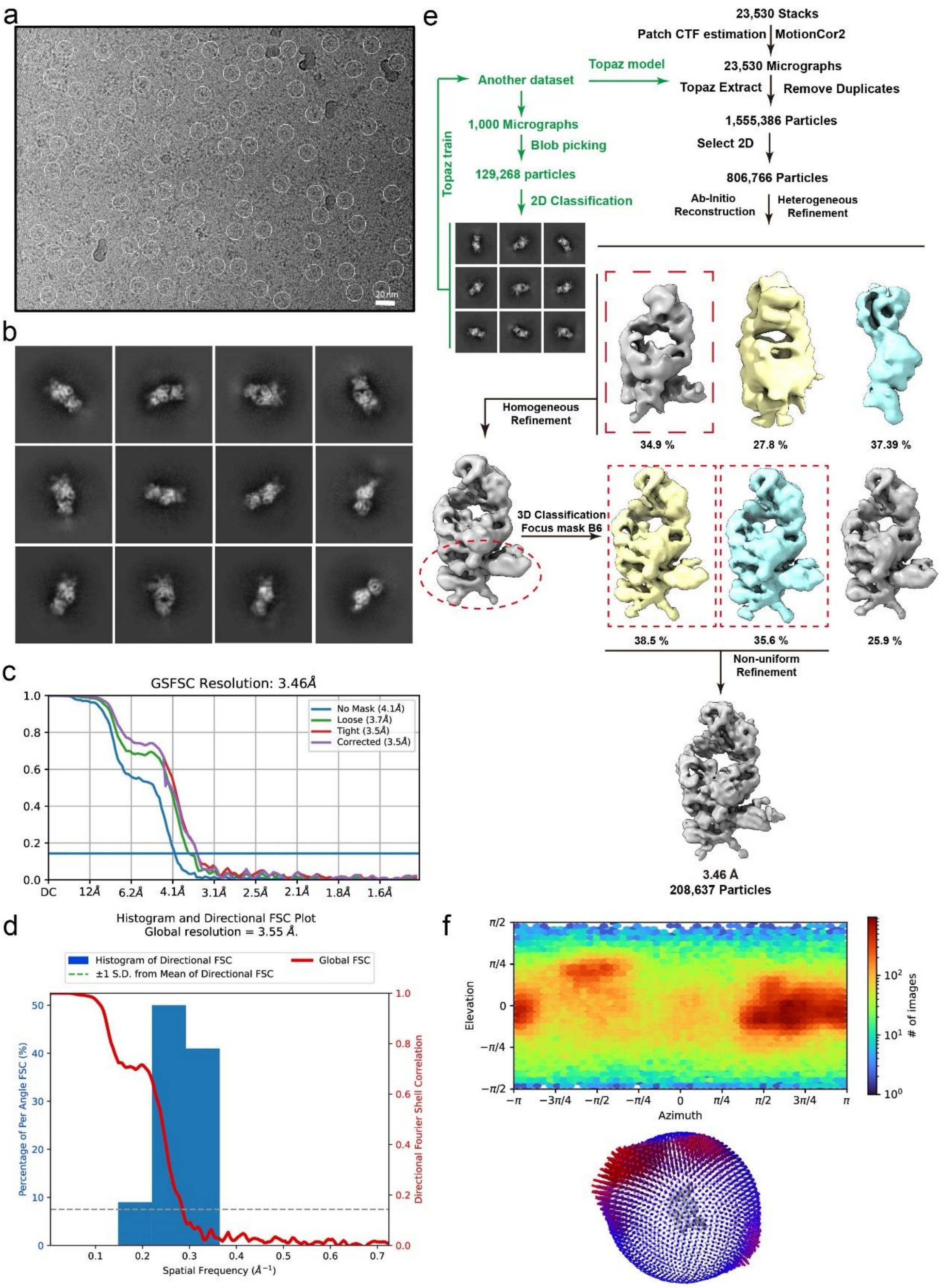

Supplementary Figure 4 Flow chart of single-particle analysis of the hMB668 in complex with MPXV B6. a Representative cryo-EM micrograph of the hMB668/MPXV B6. **b** 2D class average images of the hMB6/MPXV B6. **c** The Fourier

shell correlation (FSC) curve for reconstruction. **d** The 3DFSC sphericity analysis using cryoSPARC. **e** A brief workflow of cryo-EM image processing and reconstruction. **f** Eulerian angle distribution of the particles used in the final 3D reconstruction.

Supplementary Figure 5

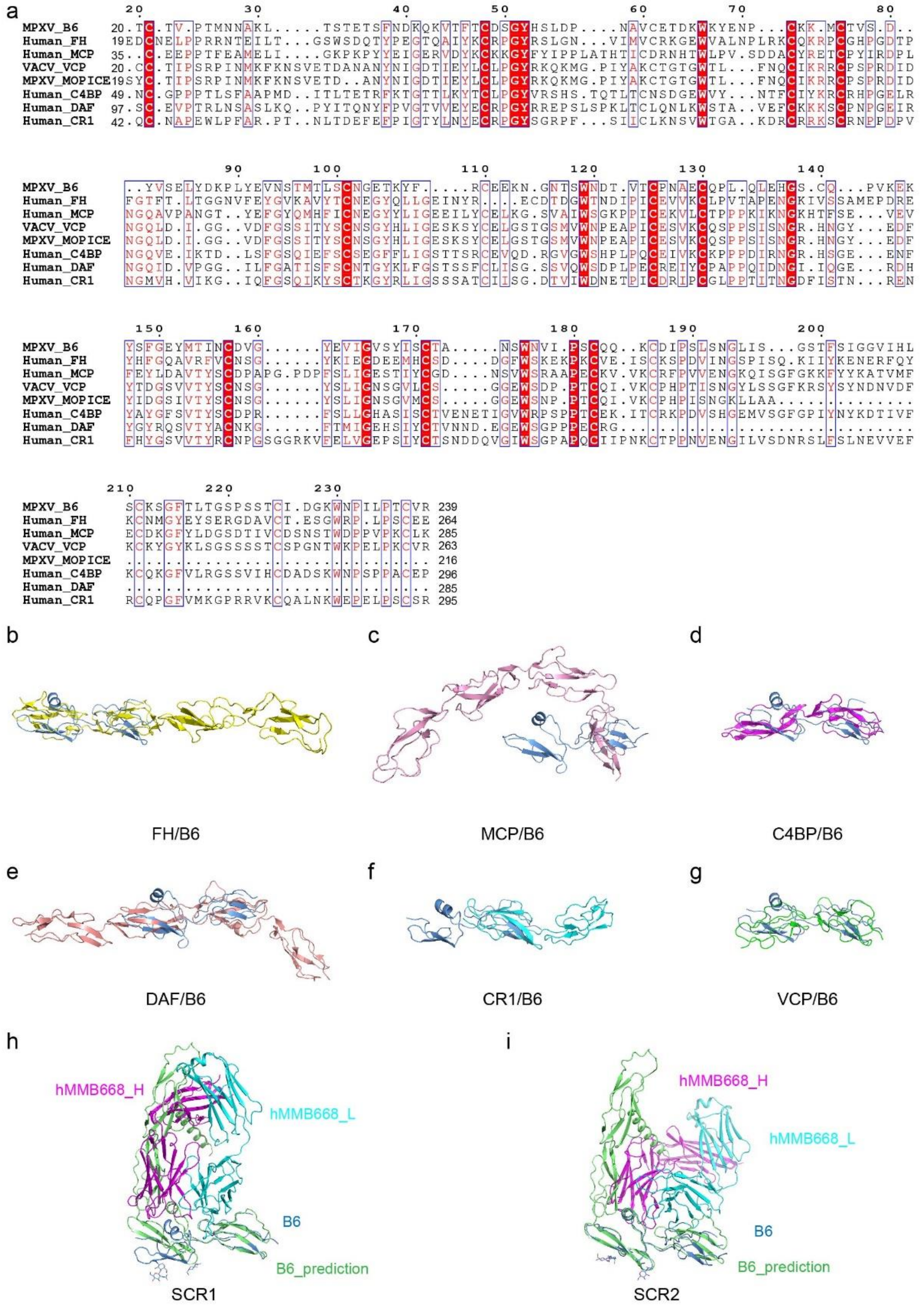

Supplementary Figure 5 Comparison of B6 with human and viral complement regulator proteins. a Sequence alignment of MPXV B6 and complement regulator proteins including human factor H (FH), human membrane cofactor protein (MCP), human C4b binding protein (C4BP), human decay-accelerating factor (DAF) and

human complement receptor 1 (CR1), as well as VACV complement control protein (VCP) and MPXV inhibitor of complement enzymes (MOPICE). **b-g** Superimposition of MPXV B6 structure onto human FH (yellow) (**b**), human MCP (pink) (**c**), human C4BP (magenta) (**d**), human DAF (wheat) (**e**), human CR1 (cyan) (**f**) and VACV VCP (green) (**g**). B6 was colored in blue. **h** and **i** Superimposition of the SCR1 (**h**) or SCR2 (**i**) in the predicted B6 structure onto the structure of B6 in complex with hMB668.

Supplementary Figure 6

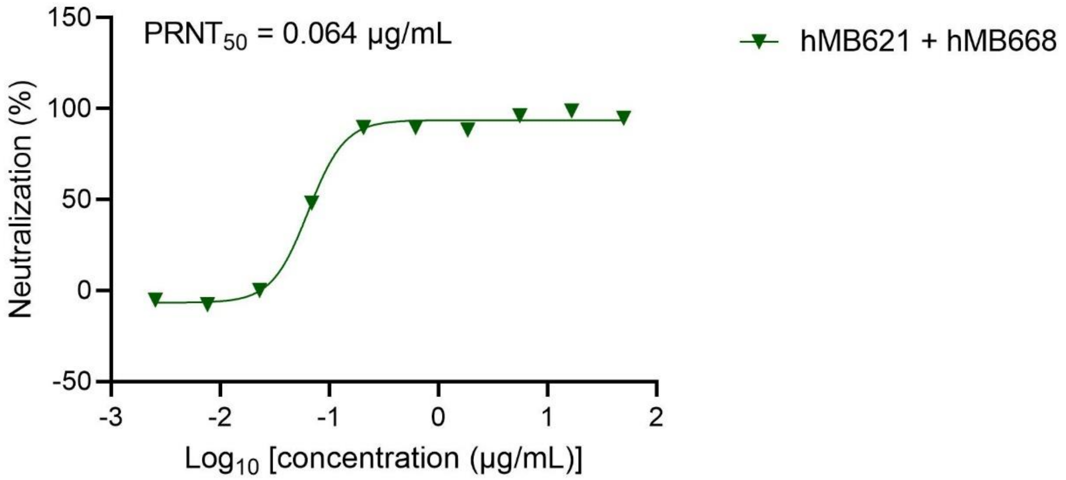

Supplementary Figure 6 Synergetic neutralizing activity of hMB621 and hMB668 against VACV. Synergetic neutralizing potency of D21 and D68 against EEV of VACV with complement were tested using a PRNT. The assay was performed twice. Representative result of two independent experiments was shown.

Supplementary Table 1 Cryo-EM data collection, refinement and validation statistics

	MPXV B6-hMB668
EMBD	EMD-38613
PDB	8XS3
**Data collection and processing**	
Magnification	105k
Voltage (kV)	300
Electron exposure (e–/Å^2)	60
Defocus range (μm)	-1.0 ~ -2.0
Pixel size (Å)	0.69
Symmetry imposed	C1
Initial particle images (no.)	1,555,386
Final particle images (no.)	208,637
Map resolution (Å)	3.46
FSC threshold	0.143
**Refinement**	
Initial model used (PDB code)	AlphaFold2; 4DN3
Model resolution range (Å)	Up to 3.46
Map sharpening *B* factor (Å^2)	no B factor value
Model composition	
Non-hydrogen atoms	4125
Protein residues	535
Ligands	3
B factors (Å^2)	
Protein	157.41
Ligand	50.00
R.m.s. deviations	
Bond lengths (Å)	0.005
Bond angles (°)	0.753
Validation	
MolProbity score	2.06
Clashscore	16.24
Poor rotamers (%)	0.00
Ramachandran plot	
Favored (%)	95.01%
Allowed (%)	4.99%
Disallowed (%)	0.00%
