# Supplementary material for: Two noncompeting human neutralizing antibodies targeting MPXV B6 show protective effects against orthopoxvirus infections
Source: Nat Commun. 2024 May 31;15:4660. doi: 10.1038/s41467-024-48312-2 (PMC11143242; doi:10.1038/s41467-024-48312-2)
Supplement: Supplementary file 3 — Description of Additional Supplementary Files [file 41467_2024_48312_MOESM3_ESM.docx]

Supplementary data 1 legend:

The sex, age range and vaccination status of all participants are shown.
