# Supplementary material for: Two noncompeting human neutralizing antibodies targeting MPXV B6 show protective effects against orthopoxvirus infections
Source: Nat Commun. 2024 May 31;15:4660. doi: 10.1038/s41467-024-48312-2 (PMC11143242; doi:10.1038/s41467-024-48312-2)
Supplement: Supplementary file 5 — Reporting Summary [file 41467_2024_48312_MOESM5_ESM.pdf]

nature portfolio

Corresponding author(s): Qihui Wang

Last updated by author(s): Apr 8, 2024

Reporting Summary

Nature Portfolio wishes to improve the reproducibility of the work that we publish. This form provides structure for consistency and transparency in reporting. For further information on Nature Portfolio policies, see our Editorial Policies and the Editorial Policy Checklist.

Statistics

For all statistical analyses, confirm that the following items are present in the figure legend, table legend, main text, or Methods section.

n/a	Confirmed	
☐	☒	The exact sample size (*n*) for each experimental group/condition, given as a discrete number and unit of measurement
☐	☒	A statement on whether measurements were taken from distinct samples or whether the same sample was measured repeatedly
☐	☒	The statistical test(s) used AND whether they are one- or two-sided<br>*Only common tests should be described solely by name; describe more complex techniques in the Methods section.*
☒	☐	A description of all covariates tested
☒	☐	A description of any assumptions or corrections, such as tests of normality and adjustment for multiple comparisons
☐	☒	A full description of the statistical parameters including central tendency (e.g. means) or other basic estimates (e.g. regression coefficient) AND variation (e.g. standard deviation) or associated estimates of uncertainty (e.g. confidence intervals)
☐	☒	For null hypothesis testing, the test statistic (e.g. *F*, *t*, *r*) with confidence intervals, effect sizes, degrees of freedom and *P* value noted<br>*Give P values as exact values whenever suitable.*
☒	☐	For Bayesian analysis, information on the choice of priors and Markov chain Monte Carlo settings
☒	☐	For hierarchical and complex designs, identification of the appropriate level for tests and full reporting of outcomes
☒	☐	Estimates of effect sizes (e.g. Cohen's *d*, Pearson's *r*), indicating how they were calculated

Our web collection on statistics for biologists contains articles on many of the points above.

Software and code

Policy information about availability of computer code

Data collection	BD AriaIII (BD Biosciences), TECAN Infinite E Plex (TECAN), BIAcore 8K (Cytiva), Octet RED96 (Fortebio), ABI QuantStudio 3 Real-Time PCR system (Thermo Fisher Scientific), 300 kV Titan Krios transmission electron microscope (Thermo Fisher Scientific).
Data analysis	GraphPad Prism (version 8.0.2), FlowJo (version 10.0.7), Biacore 8K Evaluation software (version 1.1.1.7442), ForteBio Data Analysis software (version 9.0.0.10), PHENIX (version 1.20.1), Coot (version 0.9.8), Pymol (version 2.4.0).

For manuscripts utilizing custom algorithms or software that are central to the research but not yet described in published literature, software must be made available to editors and reviewers. We strongly encourage code deposition in a community repository (e.g. GitHub). See the Nature Portfolio guidelines for submitting code & software for further information.

Data

Policy information about availability of data

All manuscripts must include a data availability statement. This statement should provide the following information, where applicable:

- Accession codes, unique identifiers, or web links for publicly available datasets
- A description of any restrictions on data availability
- For clinical datasets or third party data, please ensure that the statement adheres to our policy

Cryo-EM density map and atomic coordinates have been deposited in the Electron Microscopy Data Bank and Protein Data Bank with the accession codes EMD-38613 (https://www.ebi.ac.uk/emdb/EMD-38613) and PDB: 8XS3 (https://doi.org/10.2210/pdb8XS3/pdb), respectively. The sequences of hMB621 and hMB668 have been deposited in Genome Sequence Archive (https://ngdc.cncb.ac.cn/gsa-human/) with accession number HRA006123. Source data are provided

with this paper.

Research involving human participants, their data, or biological material

Policy information about studies with human participants or human data. See also policy information about sex, gender (identity/presentation), and sexual orientation and race, ethnicity and racism.

Reporting on sex and gender	Sex and gender were not considered in the study design and data analysis.
Reporting on race, ethnicity, or other socially relevant groupings	There is no race, ethnicity, or other socially relevant groupings correlation in the experiment.
Population characteristics	A total of 50 COVID-19 convalescents aged 16-73 years in China were selected randomly. We enrolled 30 donors (15 male, 15 female) born before 1980 as group1. We enrolled an additional 20 donors (11 male, 9 female) born after 1980 as group2. The sex of participants was determined based on self-report. We used the plasma of 50 donors for binding assay and used the PBMCs of one donor for MPXV-B6-specific memory B cells isolation.
Recruitment	Study participants were recruited at Beijing Ditan Hospital, China. All participants provided written informed consent before participation in the study.
Ethics oversight	This study received approval from the Research Ethics Committee of the Institute of Microbiology, Chinese Academy of Sciences.

Note that full information on the approval of the study protocol must also be provided in the manuscript.

Field-specific reporting

Please select the one below that is the best fit for your research. If you are not sure, read the appropriate sections before making your selection.

☒ Life sciences ☐ Behavioural & social sciences ☐ Ecological, evolutionary & environmental sciences

For a reference copy of the document with all sections, see nature.com/documents/nr-reporting-summary-flat.pdf

Life sciences study design

All studies must disclose on these points even when the disclosure is negative.

Sample size	No statistical methods were used to predetermine sample size. For mice experiments, each group contained 5 mice, which meets the requirement for statistical analysis and is sufficient to give excellent technical reproducibility.<br>To evaluate the preexisting cross-reactive antibody level to MPXV B6, 50 plasma samples were used.
Data exclusions	No data were excluded from the analysis.
Replication	The mice experiment was done once with five mice in each group, which added repeatability to the experiment. The SPR and EEV neutralization assays were repeated three times with similar results. The BLI and ELISA were repeated twice, yielding consistent results.
Randomization	The animals used in this study were allocated by random individual numbering into experimental groups. For other experiments, randomization was not a relevant feature as we were applying a uniform set of techniques.
Blinding	No blinding was performed in this study. For the in vivo protective activity analysis, the data was monitored and recorded by two people at the same time.

Reporting for specific materials, systems and methods

We require information from authors about some types of materials, experimental systems and methods used in many studies. Here, indicate whether each material, system or method listed is relevant to your study. If you are not sure if a list item applies to your research, read the appropriate section before selecting a response.

Materials & experimental systems

n/a	Involved in the study	
[ ]	[x]	Antibodies
[ ]	[x]	Eukaryotic cell lines
[x]	[ ]	Palaeontology and archaeology
[ ]	[x]	Animals and other organisms
[x]	[ ]	Clinical data
[x]	[ ]	Dual use research of concern
[x]	[ ]	Plants

Methods

n/a	Involved in the study	
[x]	[ ]	ChIP-seq
[ ]	[x]	Flow cytometry
[x]	[ ]	MRI-based neuroimaging

Antibodies

Antibodies used	anti-CD3/PE-Cy5: BD PharmingenTM, Cat No. 555334, Clone No. UCHT1, Dilution: 1:20;<br>anti-CD16/PE-Cy5: BD PharmingenTM, Cat No. 555408, Clone No. 3G8, Dilution: 1:20;<br>anti-CD235a/ PE-Cy5: BD PharmingenTM, Cat No. 559944, Clone No. GA-R2, Dilution: 1:20;<br>anti-CD19/APC- Cy7: BD PharmingenTM, Cat No. 557791, Clone No. SJ25C1, Dilution: 1:20;<br>anti-CD27/Pacific Blue: Biolegend, Cat No. 302822, Clone No. O323, Dilution: 1:50;<br>anti-IgG/FITC: BD PharmingenTM, Cat No. 555786, Clone No. G18-145, Dilution: 1:20;<br>anti-His/PE: Miltenyi Biotec, Cat No. 130-120-718, Clone No. GG11-8F3.5.1, Dilution: 1:10.<br>Peroxidase-Conjugated Goat anti-Human IgG (H+L): ZSGB-Bio, Cat No. ZB-2304, Dilution:1:3000.
Validation	The above listed antibodies were used according to the manufactures' instructions. Antibodies 8A (Chen, Zhaochun et al. PNAS. 2006) and 7D11 (Su, Hua-Poo et al. Virology. 2007) have been previously described and used in binding and EEV neutralization experiments.

Eukaryotic cell lines

Policy information about cell lines and Sex and Gender in Research

Cell line source(s)	HEK293T cells (ATCC, CRL-3216), Vero cells (ATCC, CL-81), HeLa cells (ATCC, CCL-2), HEK293F cells (Sino Biological)
Authentication	None of the cell lines were authenticated.
Mycoplasma contamination	The cells were not tested for mycoplasma contamination.
Commonly misidentified lines (See ICLAC register)	None of these cells were used.

Animals and other research organisms

Policy information about studies involving animals; ARRIVE guidelines recommended for reporting animal research, and Sex and Gender in Research

Laboratory animals	6-8-week old BALB/c mice (Vital River)
Wild animals	The study not involved wild animals.
Reporting on sex	All animals are females (our experimental design was not tailored to a specific sex).
Field-collected samples	There were no field-collected samples.
Ethics oversight	The animal experiments were conducted in compliance with the guidelines and regulations of animal welfare and were approved by the Animal Ethics Committee of the Institute of Microbiology, Chinese Academy of Sciences.

Note that full information on the approval of the study protocol must also be provided in the manuscript.

Plants

Seed stocks	*Report on the source of all seed stocks or other plant material used. If applicable, state the seed stock centre and catalogue number. If plant specimens were collected from the field, describe the collection location, date and sampling procedures.*
Novel plant genotypes	*Describe the methods by which all novel plant genotypes were produced. This includes those generated by transgenic approaches, gene editing, chemical/radiation-based mutagenesis and hybridization. For transgenic lines, describe the transformation method, the number of independent lines analyzed and the generation upon which experiments were performed. For gene-edited lines, describe the editor used, the endogenous sequence targeted for editing, the targeting guide RNA sequence (if applicable) and how the editor was applied.*
Authentication	*Describe any authentication procedures for each seed stock used or novel genotype generated. Describe any experiments used to assess the effect of a mutation and, where applicable, how potential secondary effects (e.g. second site T-DNA insertions, mosiacism, off-target gene editing) were examined.*

Flow Cytometry

Plots

Confirm that:

- [x] The axis labels state the marker and fluorochrome used (e.g. CD4-FITC).
- [x] The axis scales are clearly visible. Include numbers along axes only for bottom left plot of group (a 'group' is an analysis of identical markers).
- [x] All plots are contour plots with outliers or pseudocolor plots.
- [x] A numerical value for number of cells or percentage (with statistics) is provided.

Methodology

Sample preparation	Peripheral blood mononuclear cells (PBMCs) were isolated from the blood samples following the manufacturer's instructions (Dakewe Biotech) and stored in liquid nitrogen before use. Thawed PBMCs were incubated with His-tagged antigen and subsequently stained with anti-CD3, anti-CD16, anti-CD235a, anti-CD19, anti-CD27, anti-IgG and anti-His antibodies. After washing, antigen-specific memory B cells were sorted.
Instrument	BD AriaIII
Software	FlowJo 10
Cell population abundance	After cell sorting, the collected cells were subjected for RT-PCR to determine the genes of the IgG. Thus, we did not test the relevant cell populations within post-sort fractions.
Gating strategy	For the isolation of antigen-specific memory B cells, we used the unstained PBMCs as control to gate cells.

- [x] Tick this box to confirm that a figure exemplifying the gating strategy is provided in the Supplementary Information.
